# Supplementary material for: Molecular engineering of organic semiconductors enables noble metal-comparable SERS enhancement and sensitivity
Source: Nat Commun. 2019 Dec 3;10:5502. doi: 10.1038/s41467-019-13505-7 (PMC6890673; doi:10.1038/s41467-019-13505-7)
Supplement: Supplementary file 1 — Supplementary Information [file 41467_2019_13505_MOESM1_ESM.pdf]

Supplementary Information

Molecular Engineering of Organic Semiconductors Enables Noble Metal-Comparable SERS Enhancement and Sensitivity

Demirel et al.

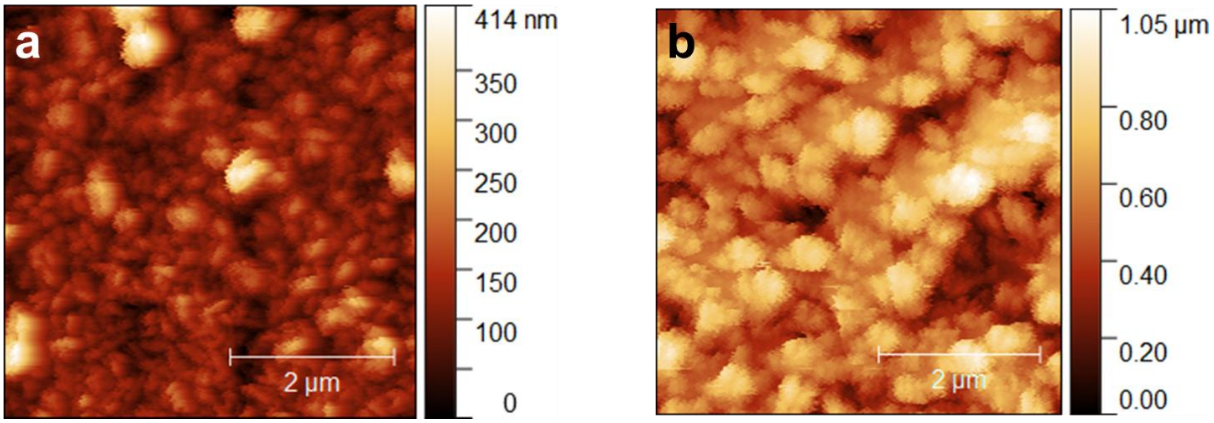

Supplementary Figure 1. AFM images of **DFP-4T** (1.1 ± 0.2 µm of thickness) (a) and **DFPCO-4T** (1.7 ± 0.4 µm of thickness) (b) films.

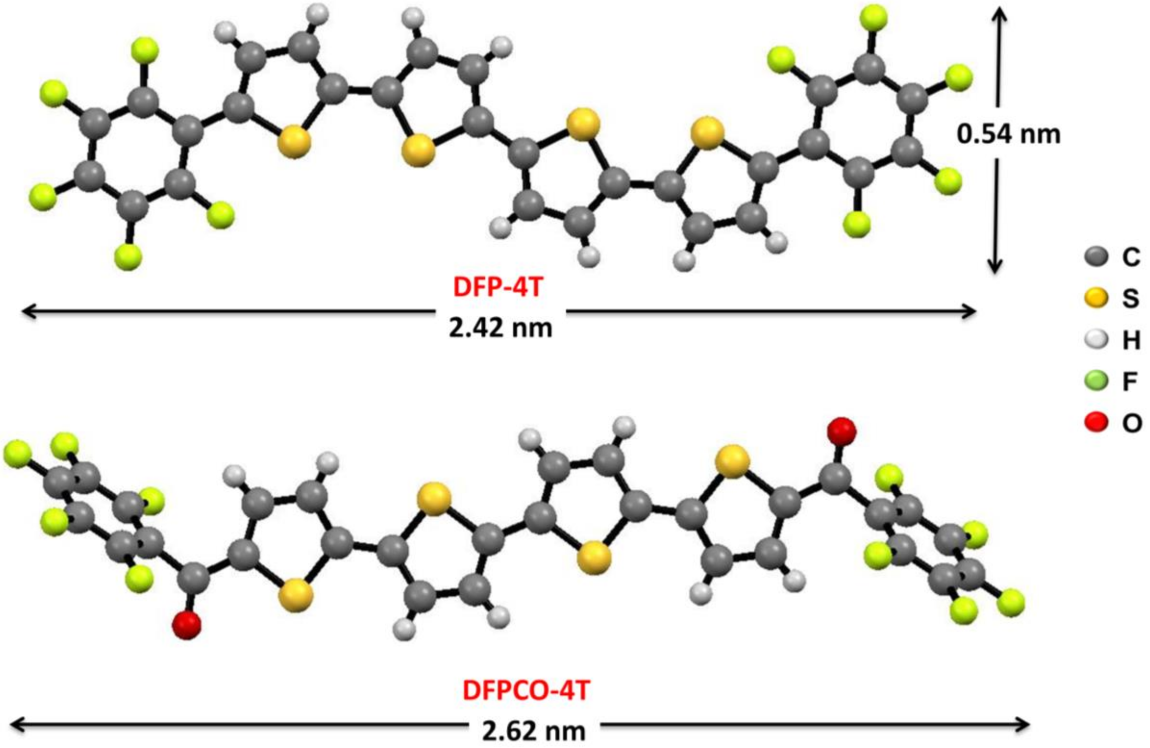

Supplementary Figure 2. Three dimensional molecular structures and lengths along the long molecular axis for **DFP-4T** and **DFPCO-4T** based on the single-crystal structure parameters.

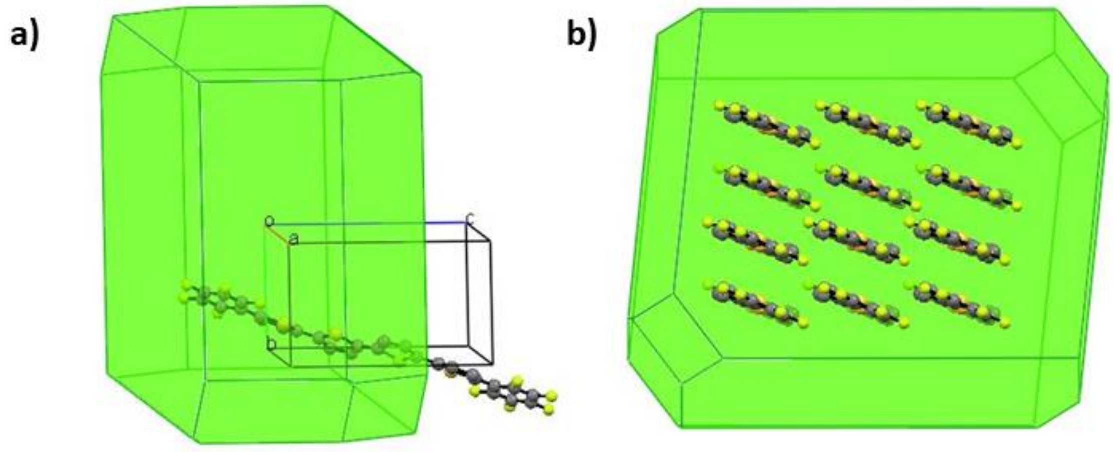

Supplementary Figure 3. BFDH (Bravais, Friedel, Donnay and Harker) theoretical crystal morphology simulated based on the single-crystal structure and view of the unit cell for **DFP-4T** (a), and molecular arrangement/packing in the 2-D crystal plane (b).

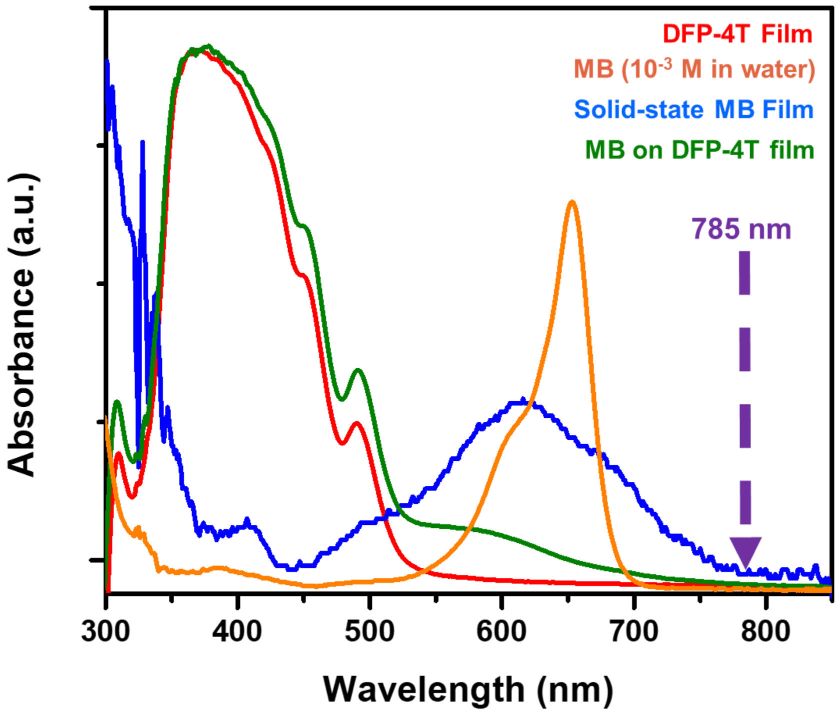

Supplementary Figure 4. UV-vis spectra of **MB** in aqueous solution (10^{-5} M) and solid state, pristine **DFP-4T** (~1.1 µm) and 10^{-5} M of **MB** deposited **DFP-4T** films. (All samples were fabricated on cleaned glass slides).

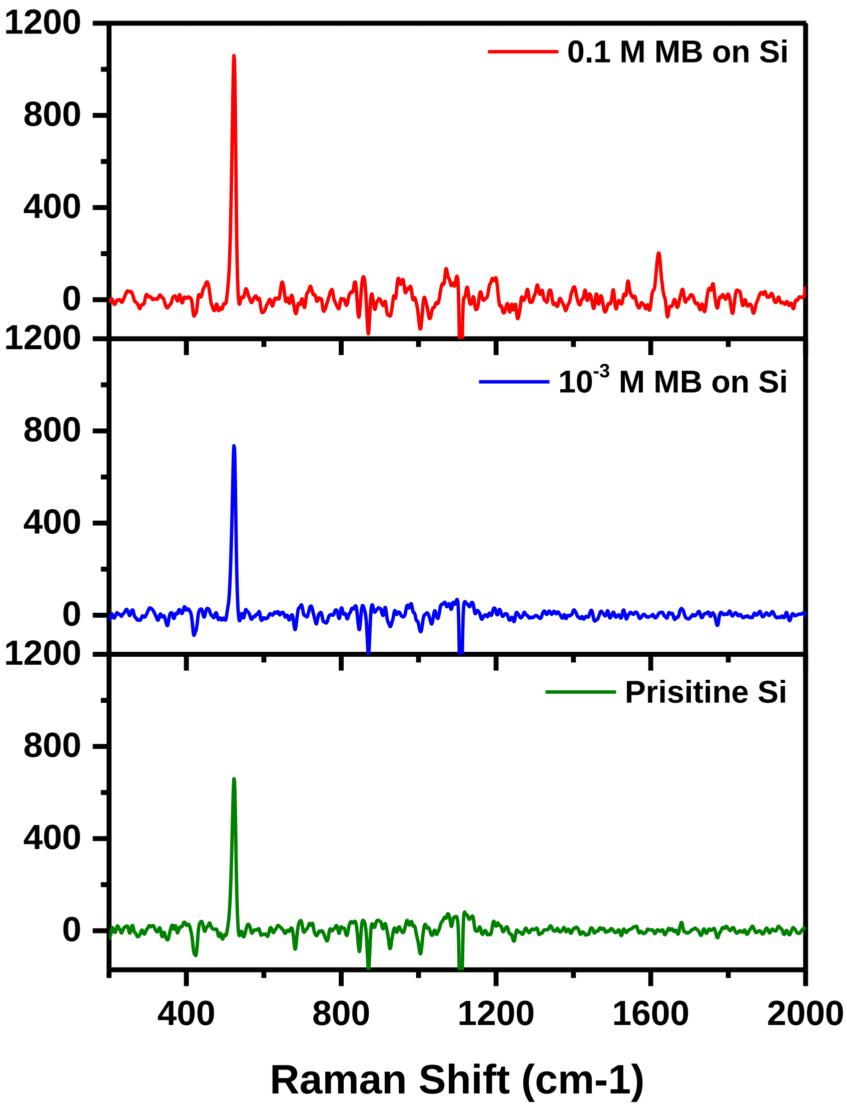

Supplementary Figure 5. SERS spectra of **MB** on reference silicon substrates.

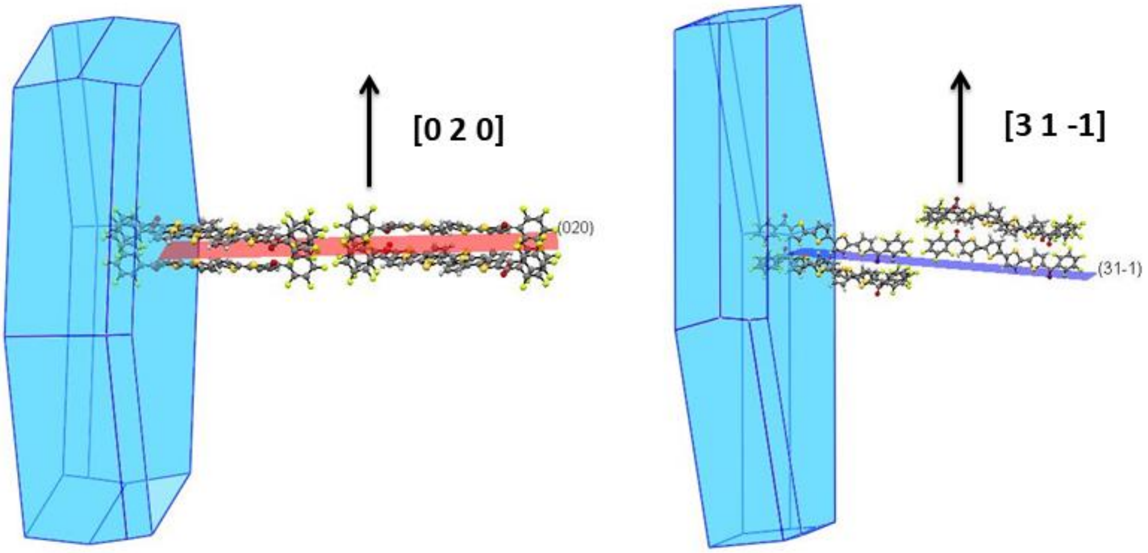

Supplementary Figure 6. BFDH (Bravais, Friedel, Donnay and Harker) theoretical crystal morphology simulated based on the single-crystal structure, and the views of molecular arrangements/packings along [0 2 0] and [3 1 -1] crystallographic directions for **DFPCO-4T**.

Substrate	HOMO Energy (eV)	CT Excited State Energy (eV)	CT Excited State Oscillator Strength	SERS Enhancement Factor (CT resonance)	SERS Enhancement Factor (1.43 eV)
DFH-2T	-7.86	2.04	0.681	4.8×10^4	0.4
DFH-3T	-7.30	1.72	0.014	9.7×10^2	0.8
DFH-4T	-7.10	1.43	0.011	2.3×10^3	2.3×10^3
DFH-5T	-6.80	1.25	0.030	3.5×10^5	25.3

Supplementary Table 1. Highest occupied molecular orbital (HOMO) energies of isolated substrate molecules; CT excited state energies and oscillator strengths; and SERS enhancement factors for the substrate-**MB** complexes at the INDO/SCI level.
